# Leveraging Electronic Health Record Technology and Team Care to Address Medication Adherence: Protocol for a Cluster Randomized Controlled Trial

**DOI:** 10.2196/47930

**Published:** 2023-07-07

**Authors:** Saul Blecker, Antoinette Schoenthaler, Tiffany Rose Martinez, Hayley M Belli, Yunan Zhao, Christina Wong, Cassidy Fitchett, Harris R Bearnot, Devin Mann

**Affiliations:** 1 Department of Population Health NYU Grossman School of Medicine New York, NY United States; 2 Department of Medicine NYU Grossman School of Medicine New York, NY United States; 3 Medical Center Information Technology NYU Langone Health New York, NY United States

**Keywords:** medication adherence, hypertension, clinical decision support, proportion of days covered, EHR, electronic health record, technology, adherence, primary care

## Abstract

**Background:**

Low medication adherence is a common cause of high blood pressure but is often unrecognized in clinical practice. Electronic data linkages between electronic health records (EHRs) and pharmacies offer the opportunity to identify low medication adherence, which can be used for interventions at the point of care. We developed a multicomponent intervention that uses linked EHR and pharmacy data to automatically identify patients with elevated blood pressure and low medication adherence. The intervention then combines team-based care with EHR-based workflows to address medication nonadherence.

**Objective:**

This study aims to describe the design of the Leveraging EHR Technology and Team Care to Address Medication Adherence (TEAMLET) trial, which tests the effectiveness of a multicomponent intervention that leverages EHR-based data and team-based care on medication adherence among patients with hypertension.

**Methods:**

TEAMLET is a pragmatic, cluster randomized controlled trial in which 10 primary care practices will be randomized 1:1 to the multicomponent intervention or usual care. We will include all patients with hypertension and low medication adherence who are seen at enrolled practices. The primary outcome is medication adherence, as measured by the proportion of days covered, and the secondary outcome is clinic systolic blood pressure. We will also assess intervention implementation, including adoption, acceptability, fidelity, cost, and sustainability.

**Results:**

As of May 2023, we have randomized 10 primary care practices into the study, with 5 practices assigned to each arm of the trial. The enrollment for the study commenced on October 5, 2022, and the trial is currently ongoing. We anticipate patient recruitment to go through the fall of 2023 and the primary outcomes to be assessed in the fall of 2024.

**Conclusions:**

The TEAMLET trial will evaluate the effectiveness of a multicomponent intervention that leverages EHR-based data and team-based care on medication adherence. If successful, the intervention could offer a scalable approach to address inadequate blood pressure control among millions of patients with hypertension.

**Trial Registration:**

ClinicalTrials.gov NCT05349422; https://clinicaltrials.gov/ct2/show/NCT05349422

**International Registered Report Identifier (IRRID):**

DERR1-10.2196/47930

## Introduction

Low medication adherence, frequently defined as a proportion of days covered (PDC) of less than 80% [1,2], is associated with increased cardiovascular morbidity and mortality and has a prevalence as high as 30%-50% among patients with hypertension [3-5]. Assessment of adherence in clinical practice is challenging; however, as most objective measures of adherence are of limited use, electronic monitoring is expensive and challenging to scale, while pharmacy claims data are limited to certain insured patients and only available to primary care providers (PCPs) with delays [4,6-8]. However, recent uptake of electronic prescribing from the electronic health record (EHR) to pharmacies offers the opportunity for scalable, real-time measurement of medication adherence that can be used for interventions at the point of care [9]. Despite the recent integration of pharmacy medication fill data into many EHRs, their use in addressing medication adherence at the point of care has not been thoroughly evaluated [10,11].

These limitations in the assessment of medication adherence in combination with the limited PCP time due to competing demands have led to insufficient attention to medication adherence in primary care practice [12]. Prior studies have demonstrated that clinical teamlets comprising PCPs paired with medical assistants (MAs) might be able to improve screening for adherence as well as medication-taking behavior [13-15]. Furthermore, the ability of teamlets to communicate and effectively change behavior can be enhanced through tight integration into the workflow using EHRs. However, the benefits of an intervention that combines teamlets with scalable, EHR-based adherence data and integrated workflows have not been studied.

The purpose of this study is to describe the design and protocol of the Leveraging EHR Technology and Team Care to Address Medication Adherence (TEAMLET) study. This cluster randomized controlled trial will evaluate the effectiveness of a multicomponent intervention on medication adherence and blood pressure control among patients with hypertension who are seen in primary care. The intervention combines a data-driven approach to automatically identify patients with elevated blood pressure and low medication adherence, team-based care to address low medication adherence, and user-centered EHR workflows. We will also evaluate the implementation of the intervention into primary care clinical sites.

## Methods

### Study Design

A 2-arm hybrid type I effectiveness-implementation pragmatic, cluster randomized controlled trial will be performed to assess the effectiveness of a multicomponent intervention on medication adherence (primary outcome) and systolic blood pressure (secondary outcome) for patients with hypertension. The clusters for randomization are primary care practices in a large health care network. The intervention is guided by the Capability-Opportunity-Motivation Model of Behavior (COM-B) framework, a parsimonious amalgamation of existing theories of behavior change, which has been proven effective for designing programs that help patients improve medication adherence [16]. Our evaluation is based on Proctor Implementation Outcomes Framework, a conceptual framework that guides the evaluation of distinct outcomes that serve as indicators of implementation success [17].

### Setting and Eligibility

This study is being conducted at primary care practices in the NYU Langone Health network. NYU Langone Health includes a diverse network of hospital-based practices, community-based practices, and community health centers in New York City and the surrounding areas. The health system includes over 60 distinct primary care practices that all use the same EHR, Epic (Epic Systems). From these practices, we will recruit 10 to participate in the study.

This study will include all adult patients seen at one of the recruited practices who have hypertension, uncontrolled blood pressure, and poor medication adherence. Specifically, we will include patients aged ≥18 years who were seen for a visit at one of the included practices in the study during the first 6 months of the commencement of the trial at that site. Patients with an active order for at least 1 antihypertensive medication will be included. To ensure patients have inadequately controlled hypertension (and mitigate variability associated with clinic blood pressure readings), we will include those with a measured clinic blood pressure ≥140/90 mm Hg on the day of the visit plus a blood pressure reading greater than or equal to 140/90 mm Hg on the previously recorded blood pressure measurement. Low medication adherence will be defined by a PDC <80% during the prior 6 months, where PDC is defined as the proportion of days that a patient had a medication from pharmacy fills while the medication had an active prescription [1,9]; for example, if a medication is initially prescribed at the start of the 6-month period, is not discontinued, and is only filled once for 90 days, the PDC would be 90/180=50%. PDC will be calculated using the algorithm in our EHR, which is based on the Pharmacy Quality Alliance measure [18]. This calculation will include adherence to all antihypertensive medications used in the prior 6 months, even if the medication was no longer active on the day of assessment. For patients on multiple antihypertensive medications, the algorithm considers a patient to be adherent on a given day only if medication fills are available for all medications on that day.

### Intervention

**Figure 1:**
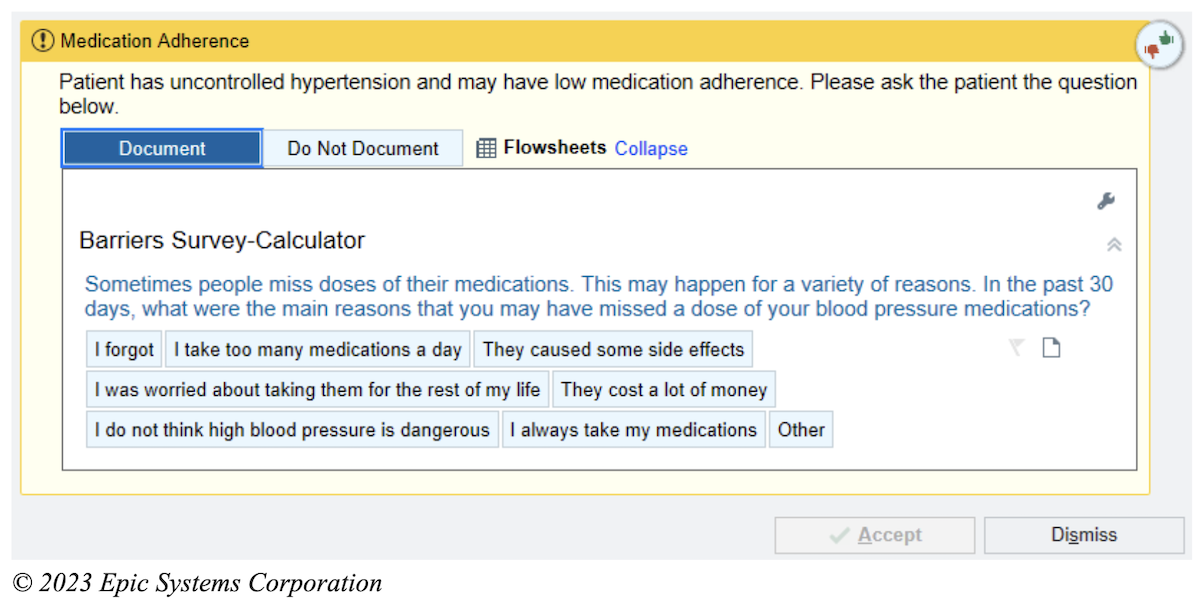
Electronic health record–based workflows for the intervention: medical assistant alerts to ask medication adherence barrier questionnaire.

**Figure 2:**
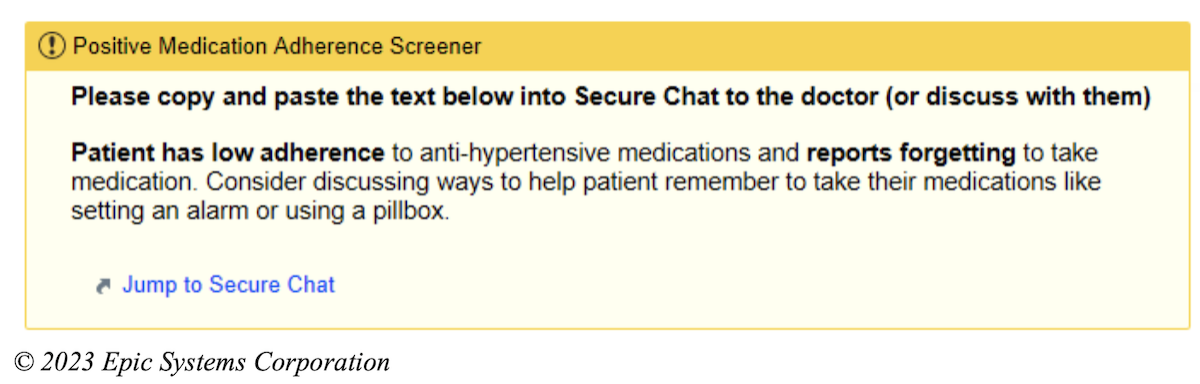
Electronic health record–based workflows for the intervention: follow-up medical assistant alert advising communication of the result to the primary care provider.

**Figure 3:**
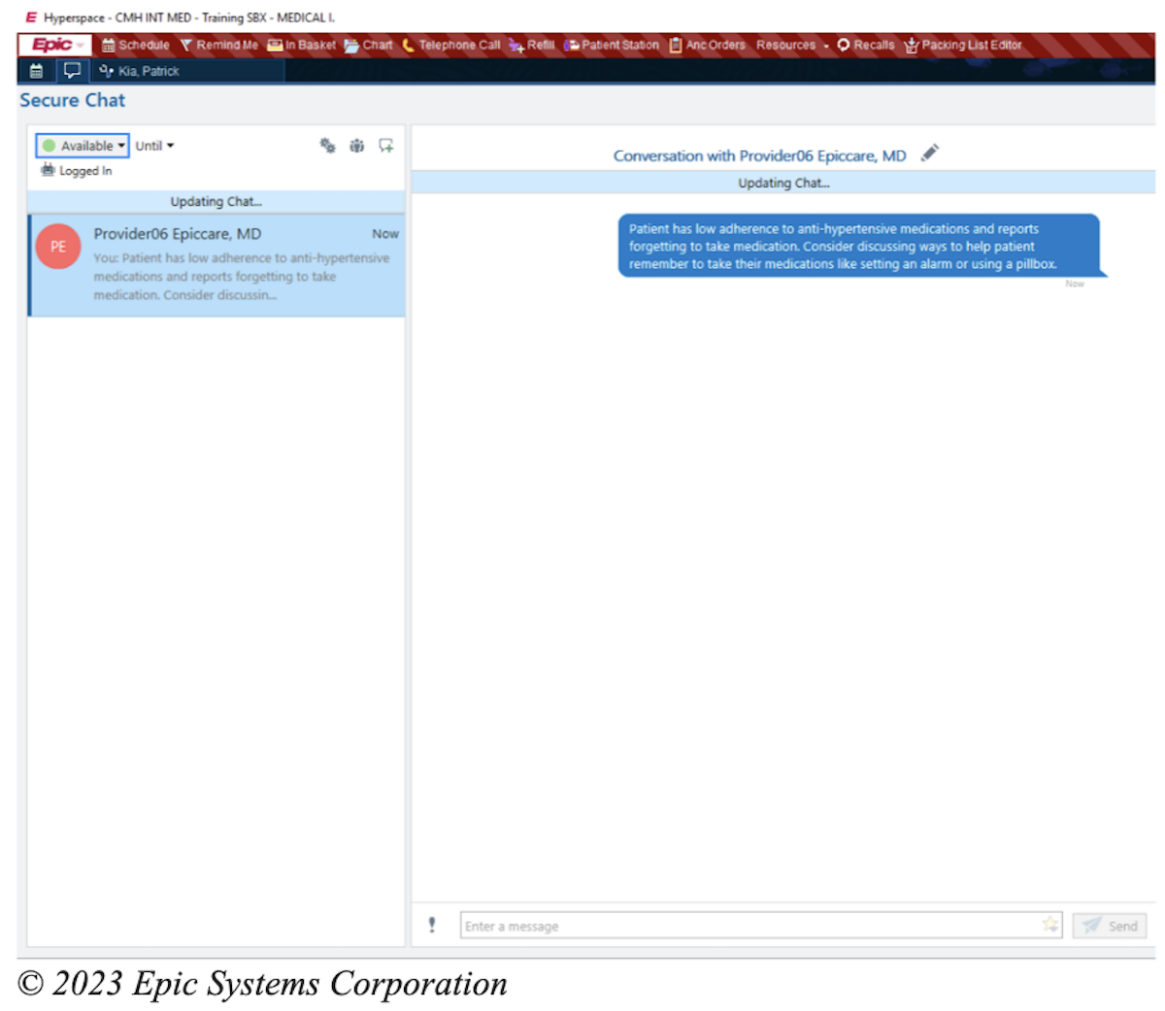
Electronic health record–based workflows for the intervention: example of secure chat to the primary care provider.

**Figure 4:**
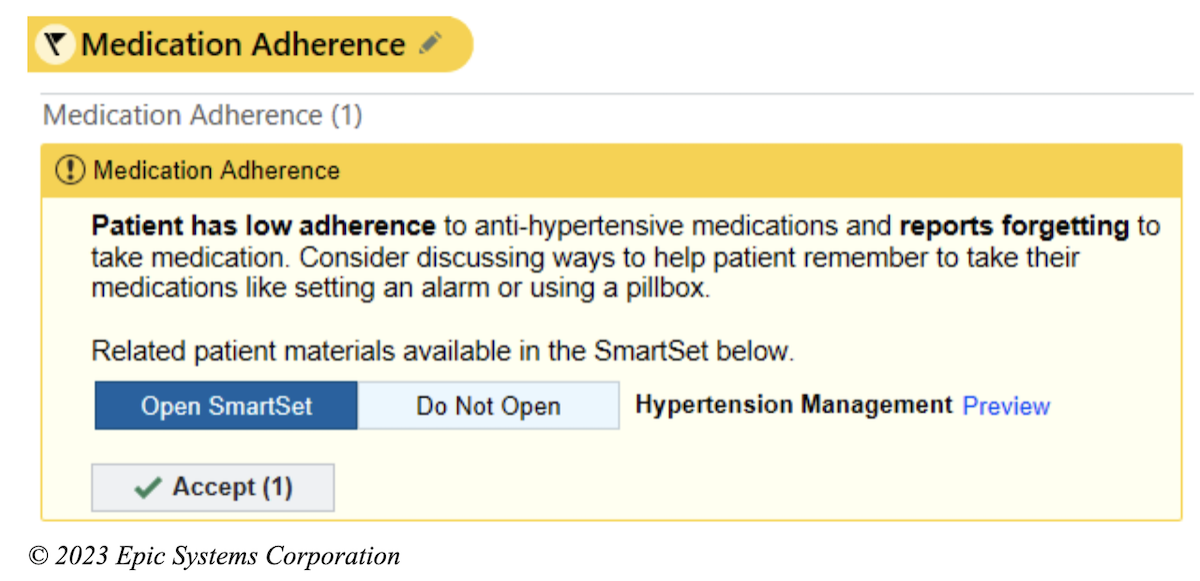
Electronic health record–based workflows for the intervention: primary care provider alert notifying of results.

**Figure 5:**
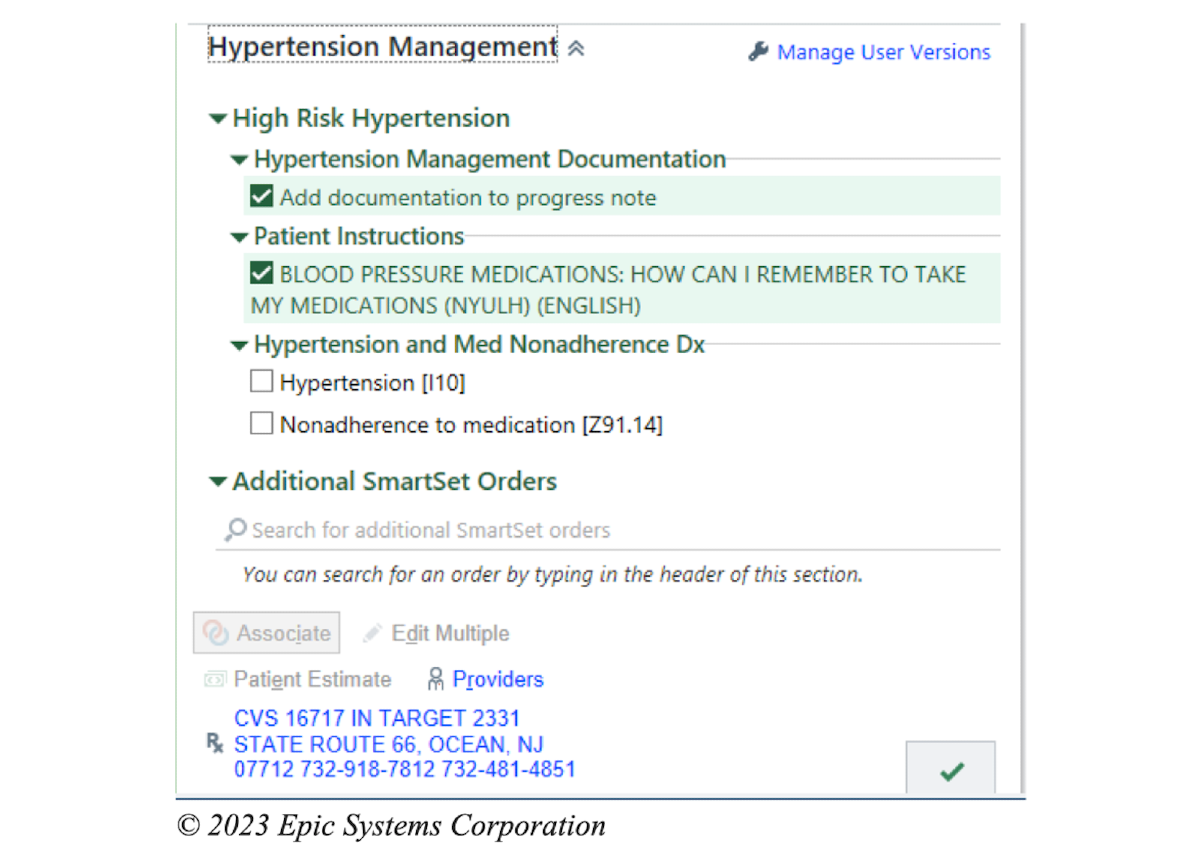
Electronic health record–based workflows for the intervention: order set available to primary care provider through the alert.

The intervention is an EHR-based workflow that consists of four components: (1) identification of patients with poorly controlled hypertension and low medication adherence at the point of care; (2) prompts for the MA to screen for important barriers to medication nonadherence; (3) alerting the PCP of low medication adherence and important barriers, with the intent of facilitating a conversation with the patient regarding medication adherence; (4) clinical decision support (CDS) to assist PCPs in addressing the barriers to medication adherence. These components have been informed through interviews and usability sessions with PCPs and MAs as well as a patient survey [19]. The process for intervention begins when the MA records the blood pressure of a given patient presenting to see their PCP. If the blood pressure is recorded as ≥140/90 mm Hg, the algorithm within the EHR will automatically screen the patient for the other inclusion criteria. If the patient meets all the criteria, that is, if their age is ≥18 years, the prior blood pressure is ≥140/90 mm Hg, they are taking an antihypertensive medication, and their PDC <80%, the MA will then receive a CDS alert to ask the patient about whether 1 of the 6 barriers contributed to missing medications (Figure 1). These 6 barriers are based on a validated questionnaire and survey of the patient population in our health system [19-21]. After responding to the question, the MA will receive an alert to communicate this result to the PCP, either through secure chat or through another method (Figure 2). The PCP then will receive the secure chat message, which will include a prompt to review the CDS related to medication adherence (Figure 3). Even if the message is not communicated from the MA to the PCP, the passive CDS will still be available to the PCP. The CDS will convey the barrier to medication adherence and provide suggestions to address the barrier (Figure 4). Additionally, the CDS will link to an order set, which will include auto documentation related to medication adherence and barriers, appropriate patient instructions, and an International Classification of Diseases, Tenth Revision (ICD-10) diagnostic code to add to the visit (Figure 5). Ultimately, our goal is for these tools to facilitate a discussion between the PCP and the patient to address barriers to medication adherence.

### MA and PCP Training

We will use a multipronged approach to train MAs and PCPs about the intervention. First, individual emails will be sent out to each MA and PCP group alerting them to the study. Included in the email will be an attachment of a tip sheet about the intervention and its components. Second, an in-person or web-based practice site meetings will be presented to include an overview of medication adherence, a walk-through of the intervention components, and suggestions for communication with patients. Third, 3 training videos that contain similar content will be built: the first containing general information about medication adherence, the second describing patient communication, and the third detailing the intervention. These supplemental videos will be accessible to intervention MAs and PCPs at any time if questions arise, although they are not required for the success of the CDS, which stands on its own. Fourth, study staff will be on-site for the first week of intervention to provide support and answer any questions during the implementation period.

### Ongoing Support

In addition to the availability of training videos that can be used at any time, site administration, PCPs, and MAs will be provided with study staff's contact information to discuss any questions or concerns, as needed. Study team members will make monthly site visits to offer additional support. Additionally, study team members will be in regular contact with site leadership to learn of any staffing changes and encourage the training of newly hired MAs and PCPs.

### Control

MAs and PCPs in the control group will receive individual emails informing them of inclusion in the study. The email will include an overview of medication adherence and will describe current tools available related to medication adherence that are available within the EHR. The first and second training videos available for the intervention group will also be accessible to control group PCPs; these videos contain general information about medication adherence and a description about communication with patients.

### Recruitment and Randomization

This study will use a practice-level cluster randomized design, with 1:1 randomization of 10 practices, resulting in 5 per study arm. We will use block randomization of 4 to 6 practices at a time with go-lives occurring approximately monthly to stagger the intervention rollout across the network.

Recruitment will be performed through email outreach to medical directors and practice managers at individual practice sites. The email will contain a brief description of the project, expectations of the practice for participation, and an offer to have a virtual meeting to present the project and answer additional questions.

### Enrollment and Follow-up Period

Patients who meet inclusion criteria will be included in the analysis if seen in one of the interventions or control primary care sites in the first 6 months after site enrollment into the trial. The patients will be evaluated for the outcome 12 months after the index clinic visit. The research support staff will support intervention implementation at each site for a total of 18 months to ensure complete 12-month follow-up for all included patients. After this time, we will observe outcomes for an additional 6 months without direct support from the research staff to assess the sustainability of the intervention.

### Clinical Outcomes

Clinical outcomes will be based on the data extracted from the EHR. Our primary outcome will be medication adherence assessed at 12 months. Adherence will be measured by PDC as a continuous variable using a lookback period of 6 months. The secondary clinical outcomes will be medication adherence as measured as a binary variable of PDC ≥80% [1,2] and clinic systolic blood pressure assessed continuously. When available, we will use the mean of the last 2 clinic blood pressure measurements within 1 year of the initial clinic visit, in order to increase reliability with multiple blood pressure measurements [22].

### Implementation Outcomes

Our implementation measures are guided by Proctor Implementation Outcomes Framework and will include adoption, acceptability, fidelity, cost, and sustainability of the intervention (Table 1). Data for our implementation measures will be obtained from both EHR data and semistructured interviews of PCPs and MAs.

**Table 1:** Description of implementation outcomes.

Outcome and measure		Data source
**Adoption**		
	For MAs^a^, we will assess: If the MAs completed the survey (yes or no) The percentage of times the MA sent a secure chat to the PCP^c^ (yes or no) Assessment of MA participation For PCPs, we will assess: If the PCP interacted with the CDS^d^ (yes or no) If the orders were placed from the order set (yes or no) If the PCP documented discussion of medication adherence with the patients. (yes or no) Assessment of PCP participation	EHR^b^ Semistructured interviews
**Acceptability**		
	We will ask MA and PCP about the acceptability of the intervention	In-person and email survey Semistructured interviews
**Fidelity**		
	Chart review for documentation of discussions MA and PCP self-reported adherence For MAs, we will assess: The percentage of times that MAs completed the survey The percentage of times the MA sent a secure chat to the PCP For PCPs, we will assess: The number of times PCP interacted with the CDS The number of orders placed from the order set The percentage who documented discussion of medication adherence with the patients.	Chart review Semistructured interviews EHR
**Cost**		
	Time spent by MAs and PCPs on intervention, multiplied by average salary	EHR Public salary data
**Sustainability**		
	Evaluate outcomes at 18 months Identify factors that influence sustainability and integration	EHR data Semistructured interviews

^a^MA: medical assistant.

^b^EHR: electronic health record.

^c^PCP: primary care provider.

^d^CDS: clinical decision support.

### Adoption

EHR data will be used to measure the adoption of the intervention by MAs and PCPs. For MAs, we will evaluate the number of MAs that completed the barriers questionnaires and the number that sent secure chat messages (measured as a yes or no dichotomous variable). For PCPs, we will assess if the order set was opened from the CDS and if the autodocumentation and patient handouts were added from the order set (measured as a yes or no variable). A qualitative assessment to understand MA and PCP participation will also be performed.

### Acceptability

We will ask the PCPs and MAs about the acceptability of the intervention during interviews and will administer a validated survey, the Acceptability of Intervention Measure at the end of the intervention [23].

### Fidelity

To assess fidelity (a metric of implementation success and quality), we will perform a chart review for a sample of eligible patients to determine the frequency of documentation of provider actions to address medication adherence, including discussions with patients and switching to alternative medications for cost or side effect issues. A weekly review will be performed at each site for the first month, then monthly throughout the study period, and provide feedback to sites if medication adherence does not appear to be addressed. The EHR data will also be used to measure the fidelity to the intervention protocol, as intended. For MAs, the percentage of barriers questionnaires completed and the percentage of secure chats sent will be measured. For PCPs, the percentage of times the order set was opened from the CDS and the number of times that auto documentation and patient handouts were added from the order set will be assessed.

### Cost

We will evaluate the cost of the intervention by estimating the additional time spent by MAs and PCPs on the intervention and then multiplying this by their average national salaries with fringe. Both self-report and data to estimate the time spent will be used. We will survey MAs and PCPs from the intervention group on how much additional time they spend with each patient for whom they receive the intervention. This value will be compared to the time the MAs spent between receiving the CDS and completion of the survey. For the PCPs, the average time spent in the EHR for intervention patients will be measured and compared to the average time on control patients. We will measure average salaries using publicly available data from the US Bureau of Labor Statistics [24].

### Sustainability

During semistructured interviews, we will ask PCPs and MAs about the opportunities to maximize the sustainability of the intervention, and the strategies to optimize continued integration of the intervention into the routine workflow. In addition, to assess the sustainability of the intervention over time, PDC will be measured longitudinally at the following time intervals: baseline, 6 months, 12 months, and 18 months (6 months post completion of intervention). Similarly, if available, blood pressure at these time points will be assessed.

### Covariate Measures

The baseline patient characteristics at the time of the initial visit with the PCP in which inclusion criteria are met will be assessed. Demographic characteristics to be collected will include patient age, sex, race, ethnicity, and insurance. Baseline clinical characteristics to be collected will include clinic blood pressure, number of medications, and comorbidities. Additionally, we will assess the number of clinic visits during the follow-up period.

### Statistical Methods

#### Sample Size

Our sample size estimate is based on the clinical outcome of medication adherence after 12 months, which is measured as a continuous variable. We plan on a 6-month enrollment period. Based on the preliminary data from primary care practices in our health system, we estimate a median of 182 eligible patients per practice during this period. Based on our prior work of having MAs perform counseling related to adherence, we anticipate a group difference of at least 20% between the intervention and the control groups [15]. Applying this effect size to our study, and assuming 80% power, 5% type I error rate (α), 30% attrition over the 18-month study period, and an intraclass correlation coefficient of 0.15 to account for clustering of patients within sites, we estimate a necessary sample size of 10 practices in the study. Calculations of sample size were estimated using a standard approach (eg, design effect) for cluster randomized trials [25].

#### Descriptive Analyses

We will summarize baseline characteristics, clinical outcomes, and quantitative implementation outcomes. Frequencies and percentages will be used to describe categorical variables, and means and SDs will be used to characterize continuous outcomes. Medians and IQRs will be reported for continuous variables that exhibit skewness.

#### Analyses of Primary and Secondary Outcomes

The analysis of all outcomes will follow the intention-to-treat principle. For the intention-to-treat approach, all participants randomly assigned to one of the 2 arms will be included in the analysis under their assigned group, regardless of compliance. The primary study outcome will be PDC, which can take on values ranging from 0% to 100%. We will develop a generalized linear mixed model with PDC as the dependent variable. The model will include a categorical indicator variable for the randomized study arm (control as reference), a variable corresponding to time (baseline as reference), and the interaction of the 2, representing the primary independent variable. The primary study end point will be at 12 months, with secondary end points measured at 6 and 18 months; these secondary end points will be used to assess sustainability. We will also adjust for any sociodemographic or clinical covariates imbalanced at baseline, as well as the number of clinic visits per patient to assess the frequency of exposure to intervention. The model will include practice-level random effects to account for clustering of patients within clinics during randomization. Prior to analyzing the data, we will compare dropout and missing data across the 2 arms and examine whether any patient characteristics were associated with the missing data. If necessary, additional analyses utilizing multiple imputation methods will be conducted.

The secondary study outcome will be blood pressure, also measured as a continuous variable using a similar generalized linear mixed model approach. Exploratory outcomes will include subgroup analyses to identify individual components of the intervention that may be influential on the clinical outcomes and other EHR-based measures.

### Qualitative Collection and Analyses

Semistructured interviews of PCPs and MAs will be conducted toward the end of the trial to identify emerging themes related to Proctor implementation outcomes. The interview guide, informed by the implementation outcomes, will consist of 10-15 questions regarding acceptability, adoption, fidelity, and sustainability, with an emphasis on barriers and facilitators to implementing the intervention. This qualitative data collection will be complemented by quantitative data collected in the study.

Qualitative analysis will include both semistructured interviews with PCPs and MAs as well as narrative reports to assess adherence. Interviews will be audio-recorded and transcribed verbatim by an outside vendor. Qualitative data will be analyzed using thematic analysis [26]. We will begin with open codes and progressively group and refine codes into categories. In addition to identifying key a priori codes based on our framework, coders will initially review a selection of interviews and develop codes to describe the content for the initial code list. The codebook will be updated and revised throughout the review process. Once all interviews are coded, the investigative team will meet to discuss and identify code clusters, relationships between codes, and common themes.

In addition, we will follow the National Institutes of Health’s best practices for mixed-methods research [27] in synthesizing quantitative results and qualitative themes by constructing a joint display for statistics-by-theme and side-by-side interpretation of the qualitative data [28]. The mixed methods approach will allow us to integrate the qualitative findings with quantitative results, providing a comprehensive understanding of the impact of the intervention, and identify the best practices for implementation.

### Ethics Approval

This study was approved by the institutional review board at NYU Langone Health (#21-00133). Verbal consent was obtained at the practice level from the medical director in addition to the ambulatory system and enterprise EHR leadership at each primary care practice. We have obtained a waiver of consent from the institutional review board at NYU Langone Health for patients included in the trial.

The Data and Safety Monitoring Board will be responsible for monitoring the study. The Data and Safety Monitoring Board will receive information specific to the intervention and control arms to evaluate the progress of the trial, including periodic assessments of data quality and timeliness; participant recruitment, accrual; and retention; participant risk versus benefit; the performance of trial sites; and other factors that can affect the study outcome.

## Results

As of May 2023, we have randomized 10 primary care practices into the study, with 5 practices assigned to each arm of the trial. Enrollment for the study commenced on October 5, 2022, and the trial is currently ongoing. We anticipate patient recruitment to go through the fall of 2023 and the primary outcomes to be assessed in the fall of 2024.

## Discussion

Medication nonadherence is a major contributor to inadequate blood pressure control [29]. However, health care providers underestimate the frequency of patient nonadherence to antihypertensive medications [30]. Relatedly, medication nonadherence is not well addressed in clinical practice: 40% of primary care visits do not include discussions about medication adherence among patients with elevated cardiovascular risk [31]. Even when these conversations do occur, they amount to a very brief discussion [32]. Lack of resources, inadequate skills, and insufficient system-level supports are the most common barriers expressed by PCPs for the lack of integration of adherence counseling into routine practice [12].

The TEAMLET intervention was developed to provide system-level support and resources to address medication nonadherence. In this study, we will conduct a cluster randomized controlled trial of this multicomponent intervention on medication adherence and blood pressure control among patients with hypertension. The intervention draws on 2 innovations to address the gaps in screening for and addressing medication nonadherence. First, the intervention uses a data-driven approach to automatically identify patients with low medication adherence and high blood pressure. Our medication adherence measure will use EHR-based pharmacy fill data, which is a scalable and reliable measure of adherence that is available in real-time clinical care [4]. To date, few studies have used these data for real-time adherence measurement and most used cases have been limited to a closed health system that includes a pharmacy [10,11]. Conversely, our study is within a health system that includes patients with various insurance providers or who are uninsured and that interfaces with hundreds of pharmacies. The second innovation of the intervention is that it relies on teamlets in primary care to address medication adherence [33]. Specifically, we will give MAs the tools to assess barriers to medication adherence and then relay this information to providers during regular clinical practice. This will allow PCPs to focus their limited time on the patient’s specific issue related to adherence and deliver tailored health coaching.

Our study has potential limitations. First, medication adherence is based on PDC from pharmacy fills; as such, it is not a direct measure of medication adherence. While PDC is commonly used in the literature to assess medication adherence, it is subject to misclassification. Nonetheless, PDC from pharmacy fill data has been shown to be well correlated with medication continuity, biomarkers, and clinical outcomes, and there is evidence that improvement in PDC leads to improvement in clinical outcomes [4,34-36]. We are also examining a clinically meaningful secondary outcome of blood pressure. Second, patient-level inclusion in the intervention and our secondary clinical outcome are based on clinic-based blood pressure measurement, which may be subject to misclassification due to issues including white coat hypertension [37]. As a result, we use a conservative definition for patients with hypertension that includes multiple high readings and a cutoff of 140/90, which is endorsed by some guidelines [38,39] but considered conservative by others [40]. Third, the study will be conducted in 1 health care system that uses 1 EHR platform. Nonetheless, Epic is the most commonly used EHR platform in the United States [41] and the intervention workflows can be generalized to most health systems and EHRs.

In this protocol, we describe a clinic-based, cluster randomized controlled trial of the TEAMLET intervention. We hypothesize that the intervention will lead to improved medication adherence among patients with hypertension being seen in primary care clinics. If shown to be true, we believe that this intervention, which is centered on data available in many EHRs and teamlets typically available in primary care settings, can be easily scaled to help address inadequate blood pressure control.

## Abbreviations

CDS: clinical decision support
COM-B: Capability-Opportunity-Motivation Model of Behavior
EHR: electronic health record
ICD-10: International Classification of Diseases, Tenth Revision
MA: medical assistant
PCP: primary care provider
PDC: proportion of days covered
TEAMLET: Leveraging Electronic Health Record Technology and Team Care to Address Medication Adherence

## Data Availability

In order to prioritize participant privacy and confidentiality, we will not be sharing any electronic health record data or qualitative data from this study due to ethical concerns regarding the sensitivity and potential reidentification risks associated with these data. However, any high-level aggregate data sets generated and analyzed in the randomized controlled trial will be made available upon reasonable request from the corresponding author.
